# Opportunities and Challenges in the Application of Bioplastics: Perspectives from Formulation, Processing, and Performance

**DOI:** 10.3390/polym16182561

**Published:** 2024-09-10

**Authors:** Daniela Negrete-Bolagay, Víctor H. Guerrero

**Affiliations:** Department of Materials, Escuela Politécnica Nacional, Quito 170525, Ecuador; daniela.negrete@epn.edu.ec

**Keywords:** bioplastic, biodegradation, additives, recycling, processability, melt processing

## Abstract

Tremendously negative effects have been generated in recent decades by the continuously increasing production of conventional plastics and the inadequate management of their waste products. This demands the production of materials within a circular economy, easy to recycle and to biodegrade, minimizing the environmental impact and increasing cost competitiveness. Bioplastics represent a sustainable alternative in this scenario. However, the replacement of plastics must be addressed considering several aspects along their lifecycle, from bioplastic processing to the final application of the product. In this review, the effects of using different additives, biomass sources, and processing techniques on the mechanical and thermal behavior, as well as on the biodegradability, of bioplastics is discussed. The importance of using bioplasticizers is highlighted, besides studying the role of surfactants, compatibilizers, cross-linkers, coupling agents, and chain extenders. Cellulose, lignin, starch, chitosan, and composites are analyzed as part of the non-synthetic bioplastics considered. Throughout the study, the emphasis is on the use of well-established manufacturing processes, such as extrusion, injection, compression, or blow molding, since these are the ones that satisfy the quality, productivity, and cost requirements for large-scale industrial production. Particular attention is also given to fused deposition modeling, since this additive manufacturing technique is nowadays not only used for making prototypes, but it is being integrated into the development of parts for a wide variety of biomedical and industrial applications. Finally, recyclability and the commercial requirements for bioplastics are discussed, and some future perspectives and challenges for the development of bio-based plastics are discussed, with the conclusion that technological innovations, economic incentives, and policy changes could be coupled with individually driven solutions to mitigate the negative environmental impacts associated with conventional plastics.

## 1. Introduction

Plastics are omnipresent materials because of their high performance in a wide range of applications, which are associated with their versatility in manufacturing, high specific mechanical properties (per unit weight), biochemical resistance, durability, and low cost [1]. However, conventional plastics have several dangerous effects on the environment, derived from their continuously increasing production, low degradation rates, the inadequate management of waste products, and pollution, both when used in manufacturing and at the end of their useful life. Global plastic production has been growing at a high rate for decades; 245 million tonnes (MT) were produced in 2008, and 368 MT in 2019 [2]. Unfortunately, most of the products obtained are intended to be single-use [3]. A clear example of this was observed during the COVID-19 pandemic. Surgical masks, face shields, syringes, gloves, medical gowns, shoe covers, and disinfectant containers were among the most used products made of plastics. Almost 1.6 MT per day of plastic waste was generated worldwide. Just a small fraction of the plastic waste produced is adequately managed. For instance, in 2013, 72% of plastic packaging was not recovered for recycling, of which 40% ended up in landfills and another 32% leaked into the environment and ended up in the oceans [4]. Under the effect of abiotic factors, discarded plastics tend to decompose slowly, and the microplastic particles that are produced (with sizes usually between 1 μm and 1 mm) persist in aquatic and terrestrial environments for very long periods [5].

**Figure 1:**
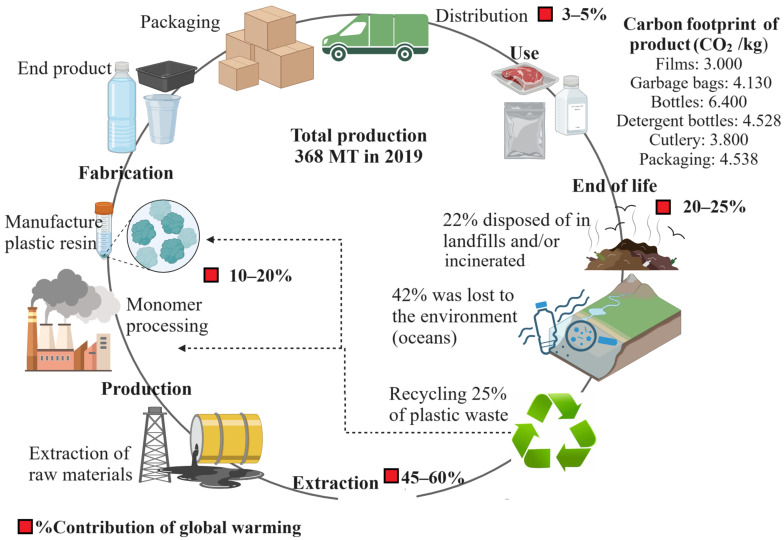
Lifecycle of plastic production and contribution of several stages to global warming [7].

There are several methods for eliminating conventional plastic wastes; the main alternative is to reuse and recycle them. Plastic recycling can be addressed by mechanical processing, chemical recycling, and energy recovery. Mechanical recycling allows plastic waste to be used as raw materials for other types of plastic products. Chemical recycling uses various chemicals to decompose plastic waste. This type of recycling involves the depolymerization of the constituent monomers through hydrolysis, alcoholysis, glycolysis, ammonolysis, pyrolysis, hydrogenation, and gasification [3]. The third alternative involves incineration to produce energy by burning plastics, which also produces extensive amounts of carbon dioxide, affecting the environment [6]. Furthermore, it should be considered that plastics can only be recycled to a limited degree because they lose various properties that affect their structure [6]. Figure 1 shows the processing stages of plastic, and its final disposal. Note that in 2018, it was estimated that 25% was recycled, 22% was disposed of in landfills or incinerated, and 42% was lost in the environment, for example, in the ocean or uncontrolled landfills [3]. As a measurement of the environmental impact of conventional plastics, their contribution to global warming can be considered. In this case, crude oil extraction causes the greatest impact, with greenhouse gas emissions ranging between 45 and 60%. End-of-product management follows at 20–25%, and finally, the manufacturing process accounts for 10–20%. These percentages collectively contribute 50% to the impact of global warming [7].

The problems associated with conventional plastics have motivated the formulation of several strategies to mitigate the tremendous impacts observed. These strategies include the development of new plastics that can replace the traditional ones or at least reduce their use in some applications [8], the improvement of product design to minimize waste [9], the creation of new and innovative alternatives for plastics recycling [10], and the deployment of public policies (mostly bans and pricing mechanisms) [11]. A converging idea involves the use of plastics within a circular economy, minimizing environmental impact, increasing cost competitiveness, and with high efficiencies along the product lifecycle. For this, it is desirable to use widely available, low cost, easy to process, renewable raw materials and residues, increase recycling, and use recycled plastics for new products. It is also necessary to produce biodegradable plastics that will be easy to biodegrade (complete digestion without special facilities), aiming to replace the petroleum-based, non-biodegradable plastics currently used, mainly in the single-use segment, moving beyond niche markets to reach large markets with demanding applications. Bioplastics represent a sustainable alternative, aligned with the paradigm described.

Biologically based plastics are derived from biological materials, that is, those originating in plants (cellulose, lignin, hemicellulose, pectin), bacteria, and algae, among others. They can be obtained using renewables and residues, and the products made of them can be expected to be biodegradable. Many bioplastics also exhibit mechanical and thermal properties similar to those of conventional plastics [12]. However, despite having almost a century of development, their growth has been slow, and their industrialization is still in the early stages. The global production of bioplastics in 2019 was 2.11 MT, which represented only 0.6% of the total production of plastics, with the expectation that in 2025 it will reach a figure of around 2.89 MT. The origin of this low production lies in several disadvantages, including high production costs, unstable or low mechanical and thermal properties and difficult processing, compared to conventional plastics [13]. Additional factors include their limited availability, lack of information about the products, biodegradation in the natural environment, and the problems associated with recycling and its high cost. Government initiatives are inefficient, demanding laws to be implemented both at the political and economic levels promoting the use of bioplastics, encouraging industries to effectively venture into their development [14]. Edible bioplastics, in the form of films and coatings, represent one of the innovative solutions that have emerged, intending to replace traditional packaging systems in markets mainly covered by conventional plastics [15]. This alternative provides researchers and consumers with a cost-effective option in terms of edibility, functionality, biodegradability, and compostability, making a significant contribution to the food sector [16,17]. In the same vein, bioactive compounds offer an innovative approach in food packaging and biomedical applications, accelerating, for instance, wound healing and improving the quality of life of patients. Obtaining these compounds creates new functional products that allow the development of novel materials that are cost-effective, non-toxic, highly effective, and bioactive [18].

The purpose of this review is to contribute to the understanding of the current state of development of those bioplastics aiming to replace conventional plastics. The performance of bioplastics in terms of their formulation, processing, and their mechanical and thermal properties, which should be at least comparable to those of conventional plastics, is analyzed. The challenges involved in obtaining high-performance biologically based plastics able to biodegrade under natural conditions in both terrestrial and aquatic environments are discussed. In commercial terms, the costs of bioplastics, their industrial development, and their production capacity worldwide are described, in markets with rigorous regulations established by different organizations. Finally, their recyclability is analyzed, keeping in mind that it is desirable that the material can be subjected to at least three recyclability processes before losing its mechanical and thermal properties [4].

## 2. Bioplastics and Their Formulation

**Figure 2:**
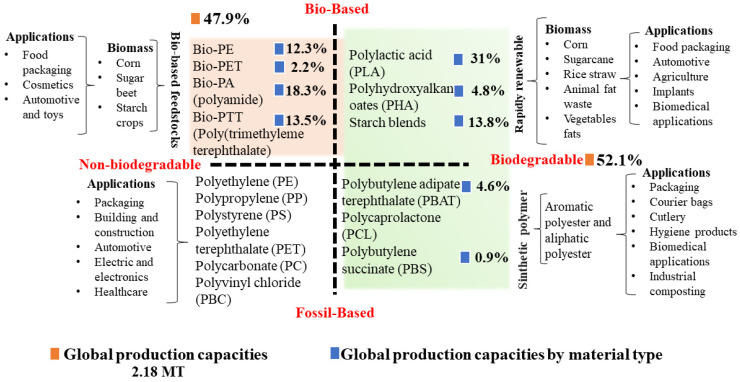
Classification and applications of bioplastics.

In 2015, the International Union of Pure and Applied Chemistry (IUPAC) defined bioplastics as bio-based polymers or polymers emitted from monomers derived from biomass [19]. Therefore, a bioplastic can be considered a biodegradable plastic, bio-based material, or both, but not all bioplastics are biodegradable, while some non-biobased plastics are biodegradable, as shown in Figure 2 [3]. If bioplastics are biodegradable, their degradation capacity depends on the environment required by bacteria or microorganisms that give rise to the enzymatic or chemical reactions produced [20]. This property is directly related to the origin of the material and its chemical structure. For example, natural polymers such as starch, cellulose, lignin, and the monomers on which their structure is based are biodegradable. However, this capacity can be lost depending on the manufacturing process of the bioplastics. That is, a chemical modification, such as polymerization, generates a loss of biodegradability. Polyamide 11, whose source of origin is castor oil, and certain types of Nylon 9, based on oleic acid, are examples of this [21].

Bioplastics are versatile and have found their way into several industries, including textiles, agriculture/horticulture, automotive and transportation, coatings, adhesives, construction, consumer electronics, and toys. Food packaging is their main application, with around 43% of the 2.18 MT of bioplastics manufactured in 2023 destined for the packaging market [22,23]. This market is dominated by conventional plastics such as polyethylene (PE), propylene (PP), polyester, polystyrene (PS), nylon, and polycarbonate (PC). Bioplastics can have a similar or better mechanical performance than the aforementioned plastics, allowing them to replace rigid food packaging products such as cutlery, containers, straws, and blown films [22]. Still, bioplastics face several obstacles; large-scale commercialization represents high production costs, and there are issues with handling and a lack of knowledge of end-of-life treatments [22,23]. The optimization of the properties of a bioplastic is linked to its formulation (type of polymer, chemical modification, additives) and processing conditions, allowing for benefits in both processing costs and properties (i.e., thermal, mechanical, oxygen and water barrier, optical characteristics, and biodegradability). This optimization could increase the possibilities of replacing or eliminating conventional plastics [23].

The development of a conventional plastic is based on its polymer matrix; however, it contains other products which are added depending on the application. This idea is replicated in bioplastics, which incorporate other compounds to modify their structure and properties. In general, the formulation must be appropriate for manufacturability and the intended application. Unfortunately, in many cases, an improvement in mechanical and thermal performance does not guarantee an increase in biodegradability. It is also highly desirable that all the constituents of a bioplastic come from renewable and biodegradable raw materials, even though several inorganic components may be used. The requirements for biodegradable plastics are established in EN 14995 [24] or ISO 17088/ASTM D6400-23 [25,26] and EN 13432 [27], which certify products that can be degraded in domestic compost, soil, and water. The specifications that must be met are a biodegradation of at least 90% in less than 6 months for any biodegradable plastic or packaging product, regardless of the type of polymer. In a composting test, material disintegration must be in 84 days, and the residual mass should be around only 10% or less. Ecotoxicity tests carried out according to the OECD Chemical Guidelines 208 require that the germination rate and plant biomass of both species (plant germination and plant growth) should be equal to at least 90% [28].

With the context depicted, the objective of this section is to describe the main additives (plasticizers, surfactants, compatibilizers, and coupling agents) used in the formulation of bioplastics, and their effect during processing and in biodegradability. Note that the inadequate formulation of bioplastics results in slow or no degradation in compost, soil, or water, increases vulnerability and exposure to water, produces a low melting point, generates toxicity (slight increase in soil acidity and microbial diversity), and low mechanical, thermal, and optical properties [21,22]. The incorporation of additives along the different processing stages allows the optimization of the physical, mechanical, and thermal properties of the material and offers the possibility of improving its thermodegradation capacity and using processing techniques with a higher productivity [29,30]. In this sense, the use of additives improves the cost–benefit relationship and ensures compatibility between different components, such as processing, manufacturing, and final user needs [31]. However, the nature and amount of additives, the compatibility between the components of a bioplastic mixture, and the polymer crystallinity, as well as the shape and size of the bioplastic products, may affect their biodegradability in specific environmental conditions [32]. Table 1 details information on the different additives used in the manufacture of bioplastics, as well as the mechanical and thermal properties obtained and their biodegradation rates.

### 2.1. Plasticizers

Plasticizers reduce fragility and crystallinity, improve durability and hardness, but they also cause a reduction in the glass transition temperature (T_g_) and decrease the elastic modulus. The plasticizers most used in the development of bioplastics are glycol, glycerol, sorbitol, fructose, sucrose, and mannose [29,44]. Glycerol and sorbitol have been reported to improve mechanical properties; however, they decrease the biodegradation capacity of potato-based bioplastics [45]. Polyethylene glycol 600 (PEG600) has also been used as a plasticizer in bioplastics of acetate cellulose obtained from *Parthenium hysterophorus*. The bioplastic films exhibited a good thermal stability and 69% biodegradation in natural conditions in 45 days. The degradation in composting and laboratory conditions were 70% and 84%, respectively [46]. The effect of adding glycerol as a plasticizer and citric acid as a cross-linker in the development of bioplastics of chitosan has also been investigated. It was observed that they increase the thickness and weight, reaching degradation in 98 days in soil, considering that the material was not subjected to any specific condition for biodegradation [47]. On the other hand, the addition of chitosan and sorbitol to thermoplastic starch had a positive effect on tensile strength and elongation at break [48].

Even though traditional plasticizers have proven to be very effective, the industry is in favor of plasticizers derived from biomass sources, due to environmental and health concerns. These bioplasticizers are expected to be less toxic, highly resistant to leaching, miscible, effective, and relatively inexpensive [49,50]. For example, Pedalium murex (PM) demonstrated its effectiveness in the development of PLA/1.0% PM film, obtaining a high tensile strength and modulus, and a slightly low elongation percentage [51]. Epoxidized soybean oil methyl ester (ESOM) can be synthesized as a plasticizer for preparing polyvinyl chloride (PVC) films. ESOM in a weight ratio of 1:2 (ESOM:PVC) delivered an increase in elongation at break (350.8%), lower tensile strength (14.21 MPa), and better thermal stability [52].

### 2.2. Surfactants

Surfactants have hydrophobic and hydrophilic groups; for this reason, they improve the manufacturing process of bioplastics because they can make polar and non-polar polymers compatible. The main function of surfactants is to increase the amount of water molecules within a bioplastic phase, thus promoting various degradation mechanisms [29]. For example, ionic liquid-based surfactants (ILBSs) have a low melting temperature which can increase the compatibilization efficiency [53]. The properties of the lipid surfactants of mannosyl erythritol B (MEL) and sodium dodecyl sulfate (SDS) were studied in biodegradable films based on cassava starch and glycerol used as a plasticizer. In this case, MEL generated flexible, water vapor-permeable, and hydrophilic films. SDS caused greater rigidity, resistance to breakage, solubility in water, lower flexibility, and permeability to water vapor [54]. ILBS was also investigated for the development of a bioplastic from polybutylene succinate (PBS) and rice starch (RS) by extrusion. ILBS significantly improved the physicochemical properties of PBS/RS mixtures by acting as a compatibilizer [55].

### 2.3. Compatibilizers

The compatibility between polymeric species, additives, and other components is crucial for the development of new bioplastics. Several investigations show that this compatibility is usually very low, which decreases mechanical, thermal, and rheological properties. This incompatibility is because one part is non-polar (hydrophobic), while the other part is polar (hydrophilic), promoting the search for compatibilizers that allow an adequate interaction [53]. Note that the compatibilizer plays a similar role to surfactants, since it gives rise to the interaction between all the molecules present [56]. The term compatibilizer may also be used for polymeric species that have intermediate affinity characteristics between the main matrix polymer and the reinforcing particles [29]. An example is the bioplastic developed from microalgae (*Spirulina platensis*) with polyvinyl alcohol as a polymer, glycerol as a plasticizer, and maleic anhydride as a compatibilizer. Different amounts of maleic anhydride were used (0 to 6 wt.%). The 6 wt.% bioplastic film achieved a tensile strength of 28 kg/cm^2^ and an elongation of 59%. Regarding the morphology analyzed, it was observed that the bioplastic film with compatibilizer had a more uniform surface [57]. Gug et al. investigated an immiscible bioplastic mixture of PLA and polyamide 11, which was compatibilized through an ester–amide exchange chemical reaction catalyzed during extrusion. An increase in both tensile strength and elongation was observed in the resulting mixture [58]. In another study, it was determined that the combination of granulated tapioca and PLA through the use of methylene diphenyl diisocyanate improves tensile strength, brittleness, and elongation at break [59].

### 2.4. Cross-Linkers and Coupling Agents

Several investigations have been carried out on the use of cross-linkers and coupling agents, such as the modification of gliadin-based films with cinnamaldehyde as a cross-linking agent. Cinnamaldehyde increased cross-linking in the films and, as a result, it decreased water absorption and weight loss, which made biodegradation difficult as it was not completed in 5 months [60]. Mihai et al. investigated mixtures of PLA, starch, and maleic anhydride. Maleic anhydride was used as a coupling agent during reactive extrusion and, as a result, increased biodegradability [61]. The biodegradation of cross-linked wheat gliadin films has also been evaluated. Gliadins were modified with different percentages of cinnamaldehyde, and this interaction increased degradation, which was completed in 4 days in compost [62]. The development of new starch-based bioplastics with double chemical and physical cross-linking network structures was investigated, with the addition of cellulose nanofibers and ZnO nanoparticles. Compared to bioplastics based on pure starch, the double cross-linking increased tensile strength, water resistance, and biodegradability [63]. Yang et al. investigated the use of a silane coupling agent (3-aminopropyl trimethoxysilane) between starch and epoxidized soybean oils. The results obtained showed greater stability, an increase in tensile strength, and other mechanical properties [64]. Nanofillers are an alternative that allows the improvement of their dispersion and distribution in the matrix. A bioplastic was developed from delignified wood (DWP) and Fe^3+^ cross-linked with tannic acid. The results showed a high tensile strength, water resistance, excellent stability, antioxidant capacity, and high biodegradability [65]. A PLA/nanosilica/epoxidized soybean oil (ESO) nanocomposite showed improved toughness and a 42% increase in elongation at break relative to PLA/nanosilica [66]. Amin et al. investigated the use of TiO_2_ nanoparticles combined with starch, vinegar, and glycerol. An improved tensile strength of the bioplastics (from 3.55 to 3.95 MPa) was observed. However, there was a decrease in the elongation at break [67].

Using the above-mentioned additives modifies the composition of bioplastics, allowing the improvement of their structural, physicochemical, mechanical, and thermal properties. This represents a promising approach for the development of strong and sustainable bioplastics with abundant and low-cost biomass sources, as well as excellent mechanical and thermal properties. The use of additives also facilitates processing by means of techniques with high production capacities such as extrusion, compression, and injection molding. This is an important step beyond the methods used at research level (e.g., solution casting), which usually allow the straightforward incorporation of various additives in film production.

## 3. Bioplastics and Their Sources

**Figure 3:**
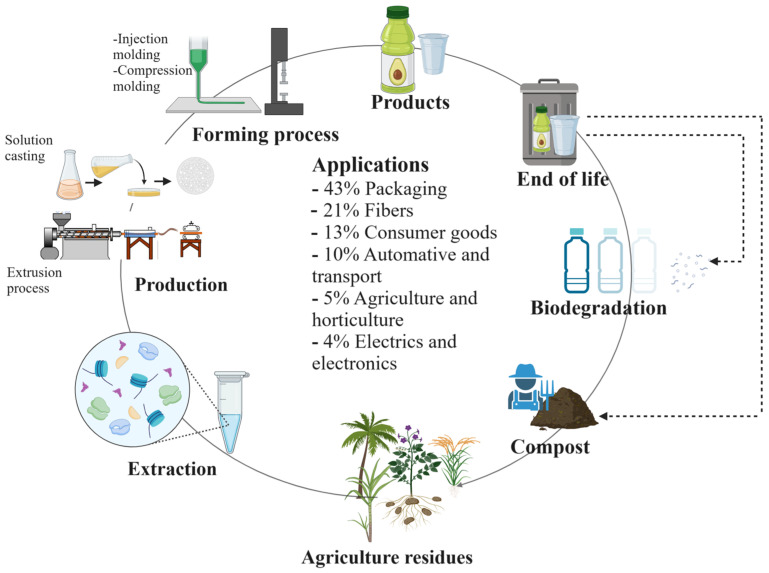
Production, molding, and applications of bioplastics from agricultural residues [74].

Biodegradable polymers can be classified into four classes: those obtained from biomass and natural resources; polymers produced by microbes through the fermentation of agricultural products; polymers that are polymerized from oligomers or monomers obtained by fermentation; and polymers derived from fossil fuels [68]. In recent years, alternative sources have been sought for producing bioplastics. Materials such as starch, cellulose, wood, sugar, and biomass in general have been investigated as substitutes for fossil fuel resources to produce bioplastics [69,70]. Agricultural waste produces around 933 MT annually worldwide [71]. Agricultural activities, food processing, alcohol, logging, and sugar production industries, among others, produce a large amount of agricultural waste, which is not properly treated and generates pollution. These agricultural wastes have been used as soil fertilizers, in paper manufacturing, and fuel production; most of them are incinerated or distributed in controlled and uncontrolled landfills [72]. However, their use in these activities is minimal, opening up opportunities for integrating them in circular economy processes to fabricate bioplastics. This section describes some of the most used sources for the synthesis of bioplastics, such as polysaccharides (starch, cellulose, chitin, and pectin). Figure 3 illustrates the extraction process of polysaccharides, their processing, application, and disposal at the end of their useful life. At this point, it is worth noting that the source of biomass, the type of plant, the species, and even its age will affect the chemical composition of its constituent polymers, directly influencing the composition of the new bioplastic to be developed [71,73].

### 3.1. Cellulose

Cellulose is the most abundant natural biopolymer on Earth, coming mainly from trees (the wood industry) and cotton [4]. Currently, the use of lignocellulosic waste as the main source of cellulose is being intensely investigated [75]. Cellulose, a linear homopolysaccharide composed of β-D-glucopyranose units linked by β-1-4 linkages, possesses a crystalline structure that renders it insoluble. This structural characteristic also contributes to its resistance to chemical and mechanical degradation. The purity of cellulose is often assessed by measuring the content of α-cellulose, while β-cellulose and γ-cellulose represent simpler sugars and other forms of carbohydrates [76]. During cellulose processing, it is important to control and choose the appropriate method for its extraction, because it is necessary to keep the cellulose intact and efficiently eliminate the other components present such as pectin, hemicellulose, and lignin. This step directly affects the performance, quality, and cost of the cellulose [75,77].

The type of treatment and manufacturing method of bioplastics is chosen according to the characteristics of the waste to be used. For example, in Malaysia, mango tree leaves were used to extract cellulose through oxidation and bleaching. The results obtained were 29.6% lignin, 33.9% cellulose, and 36.5% hemicellulose in mango leaves. The bioplastic was produced by solution casting and evaporation, with cassava starch as the polymer matrix and glycerol as a plasticizer. The results showed good flexibility and resistance. The addition of cellulose decreases the tensile strength [78]. A bioplastic based on cassava starch as a matrix and cellulose nanocrystal (CNC) from mangosteen peel as a filler was also investigated. The procedure to obtain the CNC was delignification, bleaching, hydrolysis, and sonication, then it was added in different filler proportions to the matrix. The results showed that the addition of CNC fillers to bioplastics increased the elongation at break and density [79]. In another study, cellulose obtained from *Parthenium hysterophorus* was used to synthesize cellulose acetate and then to manufacture bioplastic films. The results obtained showed a good tensile strength and elongation at break [41].

Cellulose can also be combined with other lignocellulosic materials and residues. For instance, cellulose and fiber were extracted from cocoa pod shells and sugarcane bagasse, respectively. The developed bioplastic films were divided into various cellulose and fiber concentration ratios, which were 100:0 (100% cellulose), 75:25 (cellulose/fiber), 50:50 (cellulose/fiber), 25:75 (cellulose/fiber), and 0:100 (100% fiber). The bioplastic film with the best performance was 75% cellulose and 25% bioplastic fiber [80]. Another example of the different techniques to be used for both the extraction and development of a bioplastic was in the research carried out by Syamsu et al., which was divided into four stages: cellulose extraction, synthesis of cellulose acetate, synthesis of nanofibers, and production of bioplastics by electrospinning. The electrospinning method was used for the production of cellulose acetate nanofibers and was used with cellulose acetate concentration treatments (5, 10, and 15%). Solution casting with cellulose acetate and the addition of diethylen glicol (DEG) plasticizer (0, 10, 20, and 30%) had a great performance in cellulose acetate bioplastic. The addition of 10% plasticizer had better mechanical properties, a tensile strength of 3.953 MPa, and elongation of 18.56% [81].

### 3.2. Starch

Starch is a biodegradable, biologically based polysaccharide that consists of amylose and amylopectin. This polysaccharide is synthesized through photosynthesis by most plants and is the second-largest biomass after cellulose. In 2019, starch-based polymers accounted for 21.3% of global bioplastic production [4]. The use of starch alone as a biopolymer is not viable since it has a low resistance to humidity and inadequate mechanical and thermal properties for the fusion process. Due to its biodegradability and thermal stability, starch has been used as a filler in conventional plastics, because it does not decrease the fluidity of the polymer composite. Starch is a hydrophilic molecule, which causes the poor formation of starch–polymer interfaces, due to losses in mechanical properties and low adhesion due to the hydrophobic nature of conventional plastics [77]. To overcome this, starch is processed using plasticizers (glycerol, urea, sorbitol, or glycerin), at elevated temperatures and shear forces, which improve the plasticity and thermoplastic characteristics of the polymer, obtaining thermoplastic starch (TPS) [82]. TPS exhibits a processing temperature typically ranging between 140 and 160 °C. It can soften and harden under the influence of heat and shear forces. Moreover, TPS is known for its biodegradability and shares similar transparency and odor characteristics with conventional plastics. TPS formulations with higher amylose content tend to offer superior performance in terms of tensile strength and elongation at break. However, the addition of plasticizers alters the structure and performance of thermoplastic starch, reducing its tensile strength and modulus, but improving its elongation at break [68]. Yin et al. investigated the improvement of the mechanical properties and water resistance of TPS. For this purpose, dialdehyde lignocellulose (DLC) was used through the oxidation of lignocellulose (LC) using sodium periodate. DLC-reinforced TPS composites were prepared by extrusion and injection using glycerol as a plasticizer. The highest tensile strength and elongation at break reached 5.26 MPa and 111.25%, respectively; the highest contact angle was about 90.7°, and the thermal stability increased with the addition of DLC. The addition of DLC improved the mechanical properties of the material [83].

Bioplastics based on starch as a matrix and rice straw cellulose nanocrystals as a reinforcing filler were prepared by solution casting and evaporation. This material showed a uniform dispersion of cellulose in the starch matrix; the tensile strength and modulus increased, while the elongation percentage decreased [84]. Sultan and Johari (2017) developed a bioplastic film from two biopolymers: banana peel and corn starch. The results presented five samples with different concentrations; the bioplastic with 4% corn starch reached the highest tensile strength (34.72 Pa). Better water adsorption in the 3% starch sample was observed, with an adsorption of 60.65%. The use of the plasticizer glycerol can alter the tensile strength of this bioplastic [85]. Following this line of research, the use of starch reinforced with chitosan using solution casting and evaporation showed a tensile strength of 5.19 MPa and an elongation at break of 44.6% in the 5% starch, 40% glycerol, and 20% chitosan sample, and a degradation rate of 52.1% of its initial weight after 28 days [86]. The production of starch-based bioplastics from cassava peel reinforced with microcrystalline cellulose (MCC) was investigated using sorbitol as a plasticizer. An increased tensile strength was found for the bioplastic with 6% MCC content and cassava starch plasticized with 20% sorbitol; this relation has a good adhesion between MCC and starch. However, the addition of MCC decreased the elongation at break, density, and water uptake [87].

### 3.3. Lignin

Lignin is one of the main structures of the cell wall of plants; its main source is found in the cell wall of woody trees [88]. Lignin has a degradation temperature ranging between 200 and 500 °C; that is, it decomposes more slowly. Lignin can be extracted from organic waste produced by the pulp and paper industries; however, the valorization of this product is scarce, and only 2% of the 50 million tons that is produced as waste is used in different applications [89]. The presence of various functional groups in lignin and its thermostable nature allow the search for new applications, both in the chemical industries for aromas and perfumes, and the synthesis of polymers [90]. The addition of lignin as a reinforcement of polymer matrices generates several advantages such as cost reduction, improved water absorption capacity, resistance against UV degradation, and mechanical thermo-oxidation. This is due to the antioxidant properties that lignin possesses, which allow it to act as a stabilizer due to phenolic hydroxyl groups that can eliminate free radicals. In addition, the use of plasticizers can improve the flexibility, mobility, and reduction of intermolecular forces, T_g_, and the processing temperature of the mixtures [73,77].

Several researchers have developed bioplastics using lignin as a reinforcement of a polymer matrix, to observe its behavior and generate new ecological alternatives. The results of mechanical and rheological investigations on lignin/polyethylene-vinyl acetate (EVA) composites revealed a brittle–ductile transition around a lignin volume fraction of 0.59. At higher lignin concentrations, a 36% increase in dynamic stiffness was observed by increasing load tests. The results of a thermogravimetric analysis (TGA) showed that the thermal stability of lignin increased up to approximately 55 °C as a function of EVA content [89]. A bioplastic was prepared with the macroalgae *Kappaphycus alvarezii* reinforced with lignin nanoparticles, showing that the bioplastic film with 5% lignin had an optimal improvement in almost all properties: tensile strength of 36.70 MPa, Young’s modulus of 344 MPa, elongation at break of 45%, and contact angle of 97°. The improvement of these conditions is due to the high compatibility and strong interfacial interaction between the compounds [91]. In a study performed using two types of lignin, obtained from softwood and hardwood, to manufacture composites based on PLA, the addition of lignin increased the impact resistance and thermal stability of the PLA matrix [92]. Zhu et al. investigated the development of a colorless, transparent, UV- and water-insensitive, wood-derived bioplastic by pressing delignified wood (DW) with a 0.21% lignin content and hydrophobic cellulose glass. A good wet tensile strength of 74 MPa and a water absorption of 28% were obtained [93]. Park et al. produced kraft lignin (KL) and KL compounds with PLA plasticized through the extrusion process. Furthermore, ε-caprolactone and polymeric diphenylmethane diisocyanate (pMDI) were used as KL plasticizers and coupling agents to improve interfacial adhesion, respectively. Lignin plasticization improved the dispersibility of lignin in the PLA matrix and increased the melt index. There was a slight increase in compatibility between the polymers in the tensile properties of the composites. The addition of pMDI improves the tensile strength in composites [94].

### 3.4. Chitin/Chitosan

Chitin is a biopolymer formed by N-acetyl-D-glucosamine units linked by β (1,4) bonds. The sources of chitin are the cell walls of yeasts and fungi and the exoskeletons of arthropods [77]. Chitosan is a polysaccharide that originates from chitin and is one of the most abundant natural polymers in nature [44]. Suryanegara et al. investigated the development of a bioplastic based on PLA and chitosan–ZnO and chitosan–TiO_2_ to improve antimicrobial properties, which are necessary in the food industry. The results showed that the addition of PLA to the two chitosan-based composites generates a high degradation rate and better antimicrobial properties; however, the composite is brittle, with a low tensile strength [95]. The use of starch reinforced with chitosan was investigated, using glycerol as a plasticizer. The mechanical properties were optimized, reaching a tensile strength of 5.19 MPa, and elongation at break of 44.6%. The developed bioplastic presented thermal stability, water resistance, and a degradation of 50% after 28 days [86]. The addition of chitosan to LDPE blends with maleic anhydride and tert-butyl peroxybenzoate (TBPB) as a compatibilizer and initiator was investigated. More homogeneous mixtures and a better tensile strength and elongation at break were observed. The biodegradability generated by chitosan is greater compared to low-density polyethylene [96]. The bioplastic based on starch and chitosan generated an increase in mechanical properties and a biodegradation of 80% in 14 days [97]. Another example of the efficiency of chitosan was starch–chitosan mixtures reinforced with reduced graphene oxide (rGO). The developed films had a hydrophobic surface, low solubility, and enhanced antioxidant activity [98].

Edible bioplastics illustrate the use of the biomass sources previously discussed, since they require an efficient processing and application as well as good mechanical properties, an efficient oxygen barrier, and a high-quality odor and color. Cellulose, starch, dextran, inulin, alginate, carrageenan, pectin, chitosan, mucilage, and their derivatives are some of the polysaccharides investigated for the development of edible films [99,100,101]. For example, parsley and spinach stems, rice husks, and cocoa pod shells were investigated in edible films. The results demonstrated the efficacy of these wastes, since the natural elements are transferred to the bioplastics, improving their thermophysical and mechanical properties. Torres-Leo et al. developed biodegradable coatings and films based on mango peels and antioxidant extracts of the seeds. The edible films demonstrated good barrier properties, antioxidant activity and polyphenol content increased by 18% and 60% respectively, and the gas transfer was ideal for maintaining the shelf life of the fruit [102]. In another work, agar and sodium alginate were plasticized with glycerol and water. The films developed were homogeneous, with a low roughness and high luminosity [103]. Fakhouri et al. developed composite films based on corn starch and gelatin, plasticized with glycerol or sorbitol. The results showed that gelatin increased the tensile strength, water solubility, water vapor-permeability, and thickness in the biofilms [104]. Edible films have been the subject of study due to their high capacity to replace plastics in food packaging, fulfilling the objective of preserving the environment.

The structure and properties of bioplastics open up the possibility of adding a wide variety of compounds to them, improving their stability and simultaneously providing the product with antibacterial, antioxidant, and other functional properties [105]. Bioactive agents such as antioxidants, antimicrobials, and oxygen scavengers have also been implemented in the development of bioplastics, since their use allow the generation of efficient antimicrobial materials [106,107]. For instance, Jones et al. investigated the use of proteins in bioplastics for food packaging. In this study, albumin, soybean, and whey were explored. Regarding antibacterial activity, both albumin–glycerol and whey–glycerol showed no bacterial growth after 24 h of inoculation [108]. Nanostructured edible films were prepared using mesoporous silica nanoparticles or its amino-functionalized derivative; the source was from bitter vetch seed proteins. The results showed an improvement in tensile strength and elongation at break. Furthermore, their antimicrobial and antifungal capacity makes them suitable for packaging applications due to the increase in the shelf life of various food products [109]. Zhang et al. developed films of mesoporous silica nanoparticles and cinnamon essential oil in a potato starch-based matrix. The films showed improved activity, increased tensile strength, and higher thermal stability [110]. Brites et al. extracted various extracts from rice husk, composed mainly of proteins, phenolic compounds, and carbohydrates, to be used in the formation of chitosan-based films. The results indicate that a low proportion of extracts presented a greater elongation and lower Young’s modulus compared to the chitosan film, in addition to an increase in antioxidant capacity [111]. Yong et al. investigated the incorporation of polyphenols and peptides into a soy protein/sodium alginate-based matrix. The bioplastics had good mechanical properties and a higher UV resistance. Biodegradation was around 21 days in a natural environment [112]. Bioactive compounds represent an effective strategy that facilitates entry into the food packaging market, promoting an effective circular economy. In the case of biomedical applications, the use of proteins (wheat gluten and egg white albumin) combined with bioactive agents (formic acid and oregano essential oil) has been part of the strategy implemented. The results showed that oregano oil in a wheat gluten-based matrix has applications in the controlled release of drugs or active substances [113]. In addition, the aggregation of biocide inhibits the growth of microorganisms. Nyanhongo et al. investigated the use of human serum albumin in PLA membranes to improve biocompatibility. The results indicated increased antioxidant activity, cell (osteoblast) viability, and biocompatibility [114].

The use of agro-industrial waste is proposed as a sustainable option in the development of bioplastics, complying with biodegradability criteria and reducing the associated pollution. Consistently, a great number of studies aiming to develop, make compatible, or improve the relationship between agricultural waste and other biopolymers have been carried out. Although bioplastics can be derived from a variety of raw materials, their extraction and processing can generate a significant amount of untreated waste, which is then discarded into the environment. Some extraction methods require a lot of energy, and the technologies involved are often expensive. In addition, there are the negative environmental impacts of direct and indirect land use change. For example, using primary agricultural products as raw materials can result in high production costs, reduced food availability, increased water demand, land exploitation, and the excessive use of fertilizers. Therefore, agro-industrial waste does not automatically constitute a complete solution to replacing conventional plastics [115].

## 4. Processing of Bioplastics and Biocomposites

Bioplastics are limited compared to conventional plastics, so much of the research lies in selecting, designing, and developing techniques that allow quality products to be obtained. In terms of research, the casting technique is the most widely used alternative for the manufacture of bioplastics because of its ease of manufacturing without requiring specialized equipment, resulting in materials with good mechanical and thermal properties. However, this technique demands large quantities of solvents, generating additional energy costs for solvent recovery. As it is not a continuous process, it can also have variations from one batch to another [116]. Despite this method’s disadvantages, it is still the most profitable in terms of research due to the quality of the materials obtained. In the case of edible bioplastics, most of the research is based on solution casting or layer-by-layer deposition techniques [117]. Edible cassava bioplastics were prepared by the casting technique, using glycerol as a plasticizer and modified vegetable oil to obtain a hydrophobic liquid [117]. Another edible bioplastic was prepared using gelatin, soy protein, corn starch, and papaya. The technique was developed in three steps: dilution of the corn starch, gelatin added to the solution, and glycerin as a plasticizer [118]. Jin-Kim et al. investigated the effect of a layer-by-layer electrostatic deposition coating of alginate and chitosan with grapefruit seed extract on the shelf life of shrimp. The results demonstrated a reduction in bacterial count and an extension of the shelf life of shrimp [119]. The addition of glycerol and spirulina was investigated in potato starch-based bioplastics using the solution casting technique. The results showed that glycerol and spirulina cause a decrease in tensile strength; however, elongation slightly increases [120].

**Figure 4:**
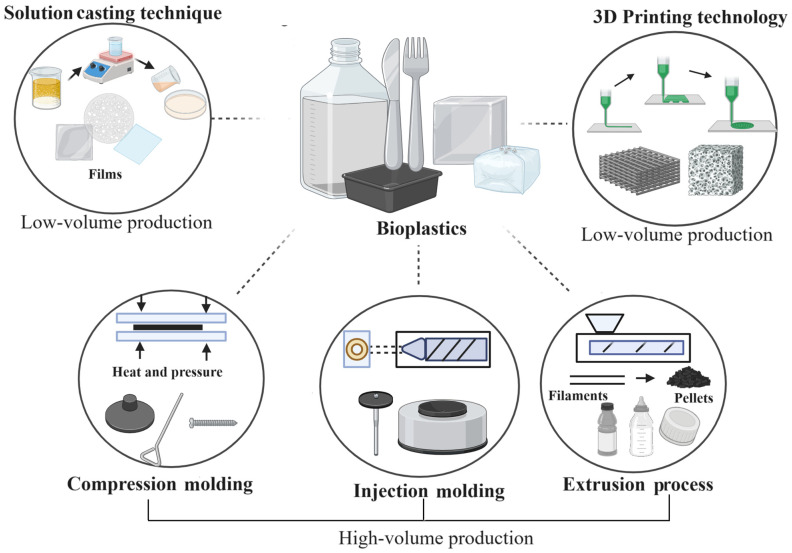
Processing of bioplastics using well-established manufacturing techniques.

On the other hand, the conventional plastics industry has defined a clear path for the selection of the manufacturing methods used to produce different parts and products for specific applications. The established techniques that generate a higher throughput in terms of overall plastic production are extrusion, injection, blow, compression, rotational molding, and thermoforming. Figure 4 shows the main processes used to obtain products based on plastics and bioplastics. Extrusion is ideal due to its high production capacity, simplicity, and profitability, although it has disadvantages such as poor distribution of raw material, high installation and maintenance costs, the high compression force required, and limitations in the type of products that can be manufactured [35,89,121]. On the other hand, compression molding also allows for high-volume production and is suitable for high-strength products, offering a low cost, short cycle times, dimensional accuracy, and impact resistance. However, it has longer process times, is not suitable for complex molds, and involves higher labor costs and limited material options [122,123]. Injection molding is efficient for high-volume production, with high accuracy, speed, repeatability, and the ability to create products in various shapes and sizes, as well as having a low cost, good corrosion resistance, and biocompatibility, but its disadvantages include the production of by-products, the need for large amounts of raw materials, and high energy consumption [124,125]. Therefore, if the petroleum-based plastics are to be replaced, it is necessary to be able to use the bioplastics being developed with these manufacturing techniques. This would avoid or reduce the need for new capital, operational, and maintenance investments, satisfying quality, productivity, and cost requirements.

### 4.1. Conventional Techniques for Processing Bioplastics

In general, bioplastics and biocomposites can be processed as conventional thermoplastics. However, processing bio-based polymers and plastics can be difficult because of rheological limitations, polymer degradation, and thermal stability issues. For instance, in the case of PLA, its highly linear molecular structure is associated with a relatively high yield of low-molecular-weight extracts and the scission of the polymer chains in processes like extrusion or injection molding. This polymer degradation decreases the viscosity and melt strength, and may result in defective parts, increased rejection rates, contamination, and corrosion of the mold surface, as well as the impossibility of obtaining the parts desired because of complete polymer degradation [126]. This could be even more problematic in processes in which the thermal degradation is higher, like those in which two steps are required (e.g., first extrusion compounding and then injection molding). For instance, Barletta et al. evaluated the thermal behavior of different PLA-based bioplastic formulations that were first compounded and then injection and compression molded. One of the formulations was not injection molded because of its thermal degradation during processing. These authors also found that the formulations compounded by extrusion showed a viscosity slightly lower than that of the PLA, which was explained by the reduction of the average molecular weight of the PLA due to the thermal degradation [127]. As in the case of viscosity, the melt flow index (MFI) may also help to assess the processability of a bioplastic. This is because the MFI determines the flow rate of the plastic material and is inversely proportional to the viscosity and molecular weight of the polymer. This parameter has been used by the plastics industry due to its low cost and easy testing [128,129]. Knowing the MFI can also help to determine the technique to be used for manufacturing with a given plastic and application. Different strategies can be used to improve the processability of bioplastics, including the incorporation of additives (as discussed in Section 2), copolymerization, blending, and the formulation of biocomposites. In the case of PLA, for instance, relatively small amounts (e.g., less than 10 wt.%) of an epoxidized chain extender were added to an injection-grade PLA. The tests performed in a twin-screw extruder showed an increase of 220% in the average molecular weight of the polymer when a 2 wt.% of the additive was used. The non-linear chain extension was efficient only at high temperatures (above 200 °C) and resulted in a better processability in terms of a much higher viscosity and improved melt strength [126]. Maleic anhydride, grafted to PLA, has also been incorporated by extrusion and used as chain extender to process PLA by injection and compression molding. Even though it has not always been found effective [126], a 2.2 wt.% added to injection-grade PLA improved the toughness of a formulation prepared with a 4.3 wt.% of talc. The flexural stiffness and strength decreased, which was explained by a decrease in crystallinity. It is also worth mentioning that in this case, a formulation without talc and a chain extender could not be injection molded after compounding, because of degradation [130].

Blending can also improve the impact resistance of bioplastics. For example, blending PBS with natural rubber resulted in higher impact strengths and elongations at break. However, the MFI of the blends prepared by means of an internal mixer decreased as the amount of rubber increased, and the Young’s modulus and tensile strength of the compression molded samples were also reduced [131]. The relatively low melt strength and the inconvenient thermo-hydrolytic degradation of bioplastics also complicates processing bioplastics by blow molding. Blending bioplastics with other polymers, rubbers, or elastomers can also help to mitigate these problems. For instance, blending polybutylene succinate-co-adipate (PBSA, MFI of 4 g/10 min at 190 °C) with an injection-grade PLA (MFI of 3 g/10 min at 190 °C) in a 1:4 ratio was defined as a good alternative to obtain a bioplastic formulation for manufacturing 187.5 mL bottles by extrusion blow molding. The PLA/PBSA blend was obtained using a twin-screw extruder and exhibited an MFI of around 59 g/10 min at 230 °C [132].

### 4.2. Natural Fiber-Reinforced Biocomposites

The formulation of biocomposites represents an alternative to overcome the fact that many bioplastics do not yet have the thermal and mechanical stability necessary to compete with conventional plastics. For this reason, several studies are focused on the effective incorporation of reinforcements instead of using additives or blending bioplastics [133,134]. Some possible reinforcements are natural fibers [135], cellulose fibrils [136], proteins, chitosan [137], and clay, among others. The use of some of these potential reinforcements was discussed in Section 3. From the point of view of processing, preparing biocomposites could be challenging, considering that two steps are usually required, first compounding by using an extruder and then molding by using a different technique. It is also necessary to take into account the limitations in the processability of the reinforcements. Existing extruders may require an appropriate modification or adaptation of the equipment and the processing parameters (e.g., shear, screw speed, and temperature), allowing the machine to process raw materials such as agro-industrial waste [138]. The bioplastics or the bio-based reinforcement to be used may require conditioning or pretreatments, such as eliminating the moisture content present. Moisture causes faster degradation, which can be observed due to the presence of holes and gaps around the filament, therefore deteriorating the performance of the material [139]. Research has also shown that treatments and modifications in reinforcements such as natural fibers allow the optimization of the quality of the fiber in the composite [140]. However, fiber wear is significant in most extrusion processes, material that is then subjected to injection or compression molding. The degree of fiber wear depends on the initial fiber length, volume fraction, and process variables (screw design, shear speed, and melt viscosity). To optimize fiber dispersion, some type of additive is often required before the final processing of the samples. However, the lack of compatibility between the natural fiber and the matrix often has more to do with differences in polarity, which can be managed with the use of compatibilizers, among other components, as described in the Section 2 [141]. Products ranging from packaging, cutlery, containers, cell phones, computers, and electronic devices to engine components are some of the main products produced by the plastic industry. However, it has now successfully made inroads into various applications such as biomedical, automotive, and electronic products and food packaging. This incursion has been successfully carried out using the processing techniques used by the conventional plastics industry, avoiding the need to change the technologies used in these companies and promoting sustainable development products. Table 2 details various techniques used in the development of bioplastic products and the different applications that have been addressed.

The reinforcements currently used in biocomposites have a wide variety of compositions, structures, sizes, and shapes. Particular attention has been received by biocomposites reinforced with nanoparticulated materials. An example of this is the research carried out with poly(adipate-co-butylene terephthalate) and thermoplastic starch (TPS) reinforced with cellulose nanoparticles using a twin-screw extruder, followed by a second extrusion to obtain films. In terms of mechanical properties, an increase of 120% in the Young’s modulus and 46% in maximum stress was obtained. The thermal behavior of the compound showed a single melting peak (T_m_) and a slight increase in T_g_, indicating a good dispersion between the phases of the mixtures [142]. PLA-based biocomposites filled with cellulose fibers and rice husk (RH) with a loading of 10 to 30% by mass were prepared by twin-screw extrusion and injection molding. Additionally, a compatibilizing agent based on PLA-g-MAH was used. The biocomposites showed an increase in rigidity and better crystallization kinetics. The composite with 30 wt.% of RH had a 32% increase in flexural modulus [143]. In a different study, MCC was incorporated into the TPS matrix using extrusion, using glycerol as a plasticizer. Additionally, films were made by compression molding. By increasing the MCC content, the tensile strength increased significantly, from 7.63 to 12.47 MPa, while the elongation at break decreased from 52% to 15% [37]. The work carried out by Aranda-García et al. presents biocomposites based on TPS, agave bagasse fibers (ABF), and PLA, in addition to glycerol. The storage, stress–strain, and flexural moduli increase with PLA and ABF content, while impact strength decreases. The T_g_ of TPS increases with the ABF content and decreases with the PLA content [144]. PLA and cellulose nanofiber (CNF) and glycerol triacetate biocomposites were prepared using a co-rotating twin-screw extruder. The results showed an increase in elongation at break and toughness from 2 to 31% and from 1 to 8 MJ/m^3^, respectively [145]. In general terms, despite the complications of the process, it can be seen that extrusion allows an increase in the storage, stress–strain, and bending moduli, as well as thermal resistance, a decrease in water absorption, and an increase in properties that justify the production of this type of composite for multiple applications. In addition, it was proven that the use of plasticizers facilitates processing because it helps the dispersion of fibers/nanofibers and provides greater fluidity within the screws. Kesari et al. developed composites based on cellulose nanofibers (CNFs) as a filler to thermoplastic starch (TPS) that caused a reduction in the MFI. The MFI for pure TPS was 2.08 g/10 min, while the addition of CNF reduced it to 1.40 g/10 min [42]. A higher melt viscosity and flow resistance due to fiber reinforcement, size, and the random position of the fibers can be observed. In addition, the strong interaction between the cellulose fibers causes the agglomeration of the fiber, which produces low mobility of the matrix molecules, and consequently, the MFI is reduced [146].

Following a different approach, fused deposition modeling (FDM) is one of the 3D printing technologies whose use has been steadily growing during the last few decades. In FDM, thermoplastics filaments are melted and deposited layer by layer until a part is completed. This additive manufacturing (AM) process, which was once basically used for making prototypes, is currently being integrated in the development of parts for a wide variety of industrial applications. This is in part because AM involves relatively simple processes, and it reduces the amount of material used and the fraction of residues generated [147]. Because of its convenient mechanical properties, melting temperature, adequate viscosity, low toxicity and biodegradable nature, PLA is the preferred material for the filaments to be used. Processing parameters such as printing orientation, infill density, and annealing temperature and time can be conveniently selected to increase the glass transition temperature and the degree of crystallinity, while decreasing the void content and microcavities between the plastic layers. This can effectively improve the mechanical and thermal behavior of the parts printed, leading to a lower hydrolytic degradation, even during aging in relatively harsh conditions such as saline environments and high temperatures [148].

### 4.3. 3D Printing Technology

On the other hand, there is a growing interest in the 3D printing of PLA-based composites reinforced with agricultural and marine particles, fibers, and residues. The use of these biocomposites offers an opportunity for the valorization of residues, aiming at the same time to reduce filament costs, increase or at least maintain mechanical and thermal performance, and improve biodegradability without compromising processability [149]. The successful production of parts by the 3D printing of biocomposites involves the adequate selection of the type, shape, and fraction of the reinforcement, as well as a careful setting of the printing parameters. Mostly single-screw extruders, but also solution casting, has been employed to mix the matrix and the reinforcements, obtaining even filaments appropriate for FDM [150]. Natural fibers and particles are among the preferred reinforcements. The particles can be easily incorporated into the PLA matrix, those of intermediate sizes (~100 µm) being the ones that offer better results. Larger particles cannot be easily used when the layer thickness is 0.1 mm or lower, and they may be difficult to disperse evenly. Smaller particles tend to agglomerate and may generate stress concentrations. As for conventionally processed biocomposites, acid and basic treatments can also be used to obtain cellulosic reinforcements, which improve the behavior of the filaments and the properties of the printed parts. A wide range of filler fractions (0.1–60 wt.%) has been used, even though the best results are typically found at low reinforcement fractions [151]. For instance, coir fiber powder was added to PLA to fabricate composite filaments that were used to print specimens. The tensile and flexural behavior of the specimens improved when 0.1 wt.% of the power was used. The mechanical properties were further improved when the specimens were annealed at 90 °C for 120 min [152]. In the case of medium reinforcement fractions, it was found that up to 20 wt.% of an extrusion-grade PLA (MFI = 6 g/10 min) could be substituted by particles of *Opuntia ficus indica* and *Posidonia oceanica*, without significantly modifying the processability of the resulting biocomposites. However, the elastic modulus and strength of these materials in tensile and flexural tests decreased as the amount of lignocellulosic fillers increased from 10 to 20 wt.%. Besides showing a potential for filament cost reduction, the composites also showed a higher hydrophilic character, which could benefit the biodegradability of the materials. [153]. A particularly interesting development was reported by Ginoux et al. [121], who used continuous extrusion AM to prepare hemp-reinforced PLA biocomposites. Hemp/PLA commingled yarns resulted in FDM specimens with a better fiber alignment, lower void content, higher homogeneity, and better tensile behavior, compared to the cases in which hemp fibers were used [154].

**Table 2:** Applications of extrusion, injection and compression molding of bioplastics and biocomposites.

By-Products	Bioplastics	Extrusion Method	Injection Molding	Compression Molding	Results	Applications
Corn starch	Starch and microcrystalline cellulose	Twin-screw extruder Temperature: At 140 °C and 140 °C	-	15 min at 140 °C under a load of 5 MPa/m^2^	Composite films containing microcrystalline cellulose are improved compared with neat starch	Packaging composites [37].
Bleached sulfite cellulose fibers from softwood	Cellulose fiber and polypropylene	Twin-screw extruder Temperature: 175–190 °C	-	160 °C at 3 MPa pressure	Pelletization and extrusion at high fiber loading caused the most severe fiber breakage	[155].
Chopped industrial hemp fiber	Cellulose acetate–cellulose acetate butyrate	Twin-screw extruder Temperature: 190–195 °C	Temperatures in zones 1-3 were 195 °C and 60 °C in the die zone. Cooling time: 40 s Pack pressure and hold pressure were 1500 and 1200 psi	-	Extrusion and injection molding processing have sufficient shear forces for the mixing of powder polymer, hemp, and liquid plasticizer	Automotive applications [156].
Cellulose nanofibers were isolated from kenaf pulp	Cellulose nanofiber and polylactic acid	Twin-screw extruder Temperature: 165–200 °C	400 bar and the mold temperature were 70 °C	-	Extrusion is a promising method for cellulose nanocomposites and achieves improved mechanical and thermal properties for PLA	Food packaging and automotive applications [43].
Cellulose acetate from pure powder free from additives	Cellulose acetate and organophilic clay	Twin-screw extruder—30 mm Temperature: 170 and 180 °C with die temperature around 195 °C.	Temperatures in zones 1–3 were 195 °C, die temperature (60–87 °C), and cooling time of 70 s. Fill, pack, and hold pressure were 8.2, 5.5, and 4.8 MPa, respectively	195 °C with pressure of 1.1 MPa for 10 min and 2.67 MPa for 5 min	Extrusion and injection molding processes increase the shear force and pressure, producing more homogeneous mixing and more clay intercalation/exfoliation	Structural applications [157].
Chlamydomonas strains microalgae	Starch-based bioplastics	Twin-screw extrusion. Temperature: 100 °C, 120 °C and 150 °C. Screw speed of 100 rpm and a mixing time of 2 min	-	-	Direct plasticization of starch-enriched microalgal biomass	Bioplastics and bioethanol industries [158].
Potato	Thermoplastic starch	Single-screw extruder—45 mm Temperature: 75–140 °C. Screw rotations: 60 to 100 rpm.	Injection speed: 70–90 mm/s Injection time: 3 s Temperature: 100 °C to 180 °C.	-	A high macro-molecular degradation takes place	Biodegradable packaging materials [159].
Passion fruit and starch	Native starches reinforced with passion fruit peel	Twin-screw extruder Automatic feeder at 5 kg/h flow rate Automatic piston liquid: flow of 2.5 L/h of plasticizers	-	At 5 ton and 90 °C for 30 s.	The extrusion process provided greater homogeneity of passion fruit incorporated in starch bioplastic	Non-food applications [138].

## 5. Recycling

**Figure 5:**
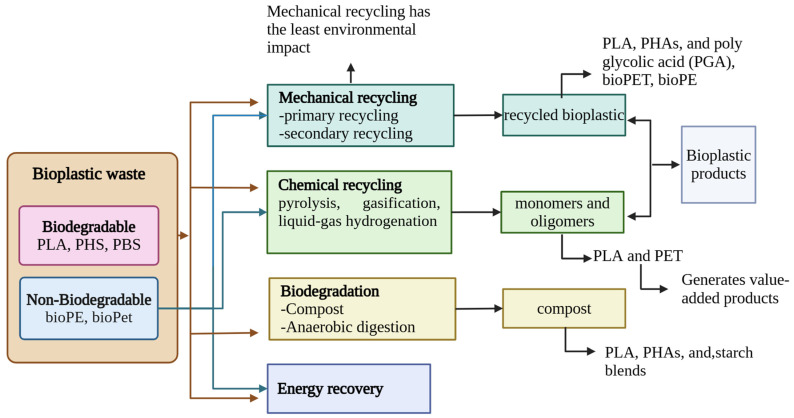
Routes of recycling of bioplastic waste [13,164].

Waste can be advantageously managed according to a hierarchy established by the European Commission, which is prevention, reuse, recycling, energy recovery, and disposal [13]. The lifecycle of each plastic material may be considered sustainable when recycling is included as a disposal option; however, this term is directly related to conventional plastics. In the case of bioplastics, biodegradation is considered the only option for the end of their useful life. This is because most people assume that all bioplastics can biodegrade in any environment [160]. But as discussed previously, sometimes biodegradation is slower, or specific conditions are required for it to take place. The quality of recycled products is affected by various factors such as cross-contamination generated between polymers at the time of collection, the presence of additives, non-polymeric impurities, and degradation. Therefore, it is necessary to combine different technologies and encourage collaboration between the population and institutions to develop effective solutions and properly manage waste [161]. The implementation of the various recycling techniques will depend on their economic viability and production capacity, which in turn will allow for a reduction in the carbon footprint [162]. The objective of bioplastics should also be to be able to recover plastic materials or monomers to reintroduce them into the lifecycle of products. This objective is met by mechanical (primary or secondary) and chemical (tertiary) recycling, followed by biodegradation in composting or anaerobic digestion. Finally, quaternary recycling through incineration is considered the last recycling option [13] (see Figure 5). In terms of recycling, thermosets and thermoplastics have significant differences. Thermosets maintain their shape and rigidity once molded, without being able to be remelted or processed again. The only option for mechanical recycling is to pulverize them for use in landfills. Thermoplastics, on the other hand, become flexible when heated, allowing them to be molded repeatedly. Although they can be reprocessed multiple times, continued recycling can lead to their degradation [163].

### 5.1. Mechanical Recycling

Assessing the optimal method for managing end-of-life bioplastic waste is crucial for its sustainable use. Each material requires an optimal end-of-life path to maximize its efficiency in the circular economy and reduce the dependence on new raw materials. Based on this, it is important to overcome the misconception that biodegradable bioplastics automatically decompose at the end of their useful life. There are processes such as mechanical and chemical recycling that offer important advantages in terms of their impact on climate change, environmental benefits, and the socioeconomic aspects compared to aerobic composting [165]. Mechanical recycling is considered a demanding process in terms of cost, energy, and labor. Primary recycling is the process used for materials that are not too contaminated and can be more easily reprocessed through extrusion. The main advantage of this recycling is that it does not require expensive equipment and is easy to handle. Secondary recycling handles all the rest of the waste that is contaminated [166]. This process involves reducing the size of the plastic to a smaller form (granules, powder, or flakes). The procedure consists of several steps ranging from collection and separation to the grinding of the material [34]. Depending on the material, the following steps will continue: agglutination, extrusion, and pelletization to distribute pellets that allow molding a new product. However, one of the main disadvantages of this process is the reduction of the thermomechanical properties of the plastics. Compared to chemical recycling, it has some advantages; no chemical additives are used, and during the process, there is a reduction in CO_2_ emissions [167,168].

Gadaleta et al. investigated the impact of cellulose acetate plasticized with triacetin (CAT) on the mechanical recycling of low-density polyethylene (LDPE). The results indicated that the presence of CAT negatively affects the reprocessing of LDPE in mechanical recycling. Therefore, the separation of this type of material is necessary due to a reduction in the quality of the recycled material [169]. Bartolucci et al. analyzed the mechanical recycling of biocomposites based on polybutylene adipate terephthalate (PBAT), TPS, and polypropylene (PP). Recycling was carried out with several extrusion cycles; the results after seven reprocessing cycles presented favorable characteristics even better than pure PP [165]. In the processing of bioplastics, the use of different additives can promote their processability; however, in terms of recyclability, it can have adverse effects. For example, plasticizers generally improve the processability and performance of PLA. As such, acrylated polyethylene glycol (acryl-PEG) was used as a reactive plasticizer for PLA. Five consecutive processing cycles were carried out, including extrusion and compression molding. A reduction in tensile and impact properties was observed, leading to embrittlement of the PLA. The deterioration of the incisions and the formation of cracks in the matrix made the material unsuitable for reuse in its initial application [170].

### 5.2. Chemical Recycling

Chemical recycling is based on converting plastic materials into smaller molecules, usually liquids or gases, which are used as raw materials for the manufacturing and production of new products. The general basis for this recycling to work efficiently is based on depolymerization, generating a profitable and sustainable method at an industrial level, providing high product performance and minimal waste [171]. Within this technique are the processes of pyrolysis, gasification, liquid–gas hydrogenation, viscosity breaking, and steam or catalytic cracking. The main advantage of chemical recycling is the possibility of treating heterogeneous and contaminated polymers [167]. However, large amounts of organic solvents are used for this procedure [172]. This type of recycling, from the point of view of bioplastics, can be employed when the quality of the material decreases below a certain threshold; the bioplastics can be chemically recycled to recover valuable monomers that could be used as building blocks for new polymers or valuable chemicals [13].

Commonly used processes for bioplastics are solvolysis, such as hydrolysis and alcoholysis, and thermal processes. From an energy perspective, solvolysis processes are cost-effective because they require less energy input than thermal processes. However, the flexibility of thermal processes, with varying operating conditions and system designs, allows for the possibility of a simpler industrial scale-up, as it is a technology already established for plastics [165]. Undri et al. performed pyrolysis tests on PLA in a microwave-assisted reactor. The result showed a relevant presence of lactides, and a positive synergistic effect of PLA with other polymers. However, it depends on the material that needs to be recovered; the combination of PLA with other plastics can generate a loss of lactides in this case [173]. Ariffin et al. investigated the thermal degradation of polyhydroxyalkanoates (PHA); this technique resulted in a successful transformation of PHA into vinyl monomers using alkaline earth compound catalysts [174]. Merchan et al. reported the thermal degradation of PBAT in the presence of halloysite nanotubes as a catalyst. The results showed a mass loss of 99.12% for pure PBAT [175].

### 5.3. Biodegradation

The biodegradation of bioplastics is influenced by factors such as temperature conditions, microorganisms, and the type of environment (e.g., terrestrial or aquatic). In addition, the chemical and physical characteristics of the bioplastic play a crucial role in its biodegradation process [176]. Conventional plastics (PE, PP, PS, PA) have strong C-C bonds or C- heteroatoms in their structure, which result in a slower degradation due to their strong stability towards chemicals, hydrolysis, temperature, light, and microbes. Bagheri et al. conducted a comparative study between biodegradable polyesters (PCL, PLA, PHB, PLGA) and non-degradable plastic (PET). The conditions were artificial seawater and fresh water. The results showed that PLGA, being amorphous, degraded 100%. This result is linked to the increase in the proportion of PLA blocks, which caused an early degradation into poly (glycolic acid) (PGA) units. Compared to the other polymers, no significant degradability was obtained [177]. In the case of PLA, its monomeric lactide component can have either an L or D chirality. This factor affects crystallinity, amorphousness, and degradation. The D forms degrade faster than the L forms [178]. Stloukal et al. studied the degradation mechanism of PLA and its composites with native or organomodified montmorillonites (MMT) under composting conditions. The addition of nanoclay improved the biodegradation of the composites. The molecular weight of PLA is an important factor, because when the molecular weight was higher, it gave way to the mineralization of PLA. PLA can be assimilated by microorganisms when the chains are shortened [179]. Molecular, crystalline, and granular structures influence the degradation capacity of starch-based bioplastics. For instance, fungal α-amylase acted as a degradation agent. The results indicated that larger molecules present in the film degrade faster; however, this does not mean that the degradation time is shorter. A more ordered structure causes the loss of its biodegradation capacity [176]. Cellulose acetate (CA) has some disadvantages in biodegradation because it does not break down easily in the environment. The main methods for its degradation include hydrolytic degradation, photodegradation, and biodegradation. Hydrolytic degradation acts on ester bonds and β-1,4 glucoside bonds. The breaking of ester bonds decreases the degree of substitution and generates acetic acid, which can accelerate degradation. The rate at which cellulose is degraded is closely related to its structure, such as the degree of substitution, crystallinity, and molecular weight [180]. Biodegradation in the soil is considered the best option due to the great diversity of microorganisms present in the medium. Several investigations have determined that species of actinobacteria are present in the soil, such as *Nonomuraea*, *Amycolatopsis*, *Streptomyces*, *Laceyella*, *Actinomadura*, and *Thermomactimyces*. The species *Streptomyces* and *Amycolatopsis* used bioplastics as a carbon source and in turn carried out their biodegradation [13]. In this type of environment, cellulose-based bioplastics have a degradation between 80 and 100% after ~100 days. In general, soil composted with bioplastics increases soil fertility and increases crop yields [181]. Biodegradation in aquatic environments is usually more complex and limited. This is due to factors such as nutrient content, temperature, pH, microbial diversity, and density, which generate a negative impact on the biodegradation of bioplastics, by reducing their biodegradation capacity. In these environments, species such as *Bacillus*, *Lepthotrix*, *Tenacibaculum*, *Pseudomonas*, *Enterobacter*, *Variovorax Gracilibacillus,* and *Avanivorax* were found [181,182]. However, depending on the type of bioplastic and the conditions of the composition of soil or water, its degradation rate can be significantly lower than desired. Another disadvantage of biodegradation is that any value embedded in the molecular structure of the polymer is lost. Instead, work must be done towards a circular economy approach, where bioplastics are recycled both mechanically and chemically to recover material value [183].

#### 5.3.1. Composting

Composting is a profitable and desirable method to eliminate bioplastic waste. Its main objective is to decompose the organic matter present and produce humus, which is used as a nutritional source for the soil and its fertilization. According to Rosenboom et al., biodegradation and composting describe the microbial digestion and metabolic conversion of polymeric material into CO_2_ and H_2_O. During this process, various physical interactions lead to fragmentation and a reduction in particle size [182]. Specific microorganisms, such as *Pseudomonaceae*, *Comamonadaceae*, and *Erythrobacteraceae*, *Streptomycetaceae*, the families *Caulobacteraceae* and *Enterobacteriaceae*, and enzymes such as N-acetyl-β-glucosaminidase, esterase, β-glucosidase, acid and alkaline phosphatase, and phosphohydrolase, are involved in microbial activity, degradation, and bioplastic decomposition [181]. Several industries are adopting composting technology as a safe and cost-effective solution for waste management. The European standard EN 13432 [27] establishes requirements for a plastic material to be considered compostable. This material must degrade by at least 90% by weight in 6 months, and it must be reduced to fragments smaller than 2 mm in contact with organic materials in 3 months [184].

#### 5.3.2. Anaerobic Biodegradation

Anaerobic biodegradation occurs in the absence of oxygen in biogas plants that are mesophilic with a temperature of 37 °C or thermophilic with a temperature of 55 °C. During this process, organic matter is converted into methane gas, carbon dioxide, water, hydrogen sulfide, ammonia, and hydrogen, giving rise to a sequence of metabolic interactions between different groups of microorganisms [185,186,187]. Factors include temperature, humidity, light, incubation time, pH, pressure, polymer structure, and the concentration of viable microbial cells. These conditions allow the efficient anaerobic degradation of bioplastics to be obtained. This process involves four main stages: biodeterioration, fragmentation, mineralization, and assimilation. The adhesion and colonization of microorganisms on the surface of bioplastics is crucial to initiate the biodeterioration process, which modifies the polymers’ physical, chemical, and mechanical properties. Microorganisms, unable to use large macromolecules, secrete enzymes. These enzymes degrade polymer chains into oligomers, dimers, and monomers, an interaction that occurs through hydrophobic interaction, where the enzymes can accommodate hydrophobic groups of the polymer in their structure. Microorganisms then assimilate the fragmented molecules for oxidation and energy production, and subsequently the oxidized components, such as carbon dioxide, nitrogen, water, and methane, are released into the environment during mineralization [188]. A typical biogas plant treating urban organic waste operates with a hydraulic retention time of 15 to 30 days under thermophilic or mesophilic conditions. Therefore, a bioplastic bag should degrade under these conditions. PHB, starch, cellulose, and pectin tend to degrade during this time [185]. Nacho et al. used batch reactors to evaluate the biodegradation by anaerobic digestion of three bioplastics and PET at mesophilic (35 °C) and thermophilic (55 °C) temperatures. In addition, two treatments with PHBV were carried out at 35 and 55 °C. The PHBV treatment at 35 °C produced the highest amount of methane (CH_4_) normalized by the volatile solids of the bioplastics. Most bioplastics produced more CH_4_ than PET [189].

The inefficient management of end-of-life products is attributed to a lack of adequate infrastructure for collection and processing, insufficient or non-existent policies, and a lack of knowledge about waste recycling, which leads to inappropriate waste sorting. Furthermore, there is a lack of commitment on the part of people to adopt recycling practices. Therefore, recycling bioplastics remains a significant challenge. Although attempts have been made to apply technologies and standards similar to those used for conventional plastics, combining both types of materials can lead to contamination both in the material and in the environment. The misperception of bioplastics as biodegradable under all conditions leads to the inappropriate disposal of these materials, as users do not seek other forms of recycling.

## 6. Bioplastic Market

Despite being on the market for a long time, bioplastics have not yet reached a competitive level comparable to that of petroleum-derived plastics. This growth in the bioplastics industry is closely related to extracting raw materials and processing polymers into final products. Fluctuations in raw material prices and demand for final products increase extraction and production costs. In addition, factors such as biodegradability issues, lack of policies, and lack of consumer awareness about the variety of products and their identification often hinder the growth of bioplastics in the market [190,191]. According to Fortune Business Insights, in 2022, the bioplastics market had a valuation of 7.56 billion dollars, and it is expected to increase to 31.66 billion dollars in 2030, exhibiting a compound rate of 20.2% annually [192]. Europe is considered one of the main bioplastic producers, with around 25% of total production. Asia has been presented as a developing power because it has the greatest production capacity. Next is North America, which is venturing into the development of bioplastics to promote environmentally friendly products [192]. The main companies responsible for this growth are BASF (Malmparken, Ballerup, Denmark), Corbion N.V. (Amsterdam, The Netherlands), NatureWorks LLC (Plymouth, MN, USA), CJ CheilJedang (Jung-Gu, Seoul, Republic of Korea), Novamont (Novara, Italy), and Tianjin Guoyun (Tianjin, China) [193]. An example of this is the Futamura Chemical Company, with its product Cellophane, a transparent film made from cellulose pulp, whose application ranges from cellophane tapes to the packaging of pharmaceutical and food products [194]. This company had a revenue of USD 11.19 billion. Novamont produces its flagship product Mater-Bi. This product is obtained through patented technologies pioneering the use of starches, cellulose, vegetable oils, and their combination [195]. The advancement in biodegradable and compostable bioplastics, for the company, represents 25 years of research and innovation and involves three main plants located in Italy [196]. The chitosan market has begun to show exponential growth. This is due to the diversity of applications to which it can be directed (e.g., biopharmaceutical, cosmetics, biotechnological and biomedical, agriculture and food and non-food industries, water treatment) [197]. The cosmetics industry in 2015 was the largest chitosan application sector in North America. Bioplastics need to meet a wide range of requirements to be able to enter the market, but depending on the application, there will be more or less effort to replace conventional plastics. In terms of market demand, consumers give more or less importance to the biodegradability of components depending on the application. For example, the food industry has high growth in the bioplastics market because the consumer looks for environmentally friendly packaging that does not cause negative effects on vegetables, fruit, or meat, among others. This also applies to healthcare [198]. However, the automotive and transportation areas seek products that last longer, and the term biodegradability is not usually considered efficient. Unlike packaging for food products, the customer looks for resistant and durable products. They then demand much higher quality standards from the polymer that bioplastics often do not meet. The demand is still moderate compared to conventional plastics, but there are sectors in which there is a specific interest in bioplastics. These sectors allow industries to venture with greater interest into the research of new bioplastics [198]. Table 3 shows various applications of bioplastics depending on their material.

It is worth mentioning that several companies worldwide have ventured into the world of bioplastics by eliminating certain packaging or products from their production chain. In general, food packaging is a leader in the use of bioplastics. Companies such as McDonald’s and Burger King have eliminated the use of certain PS- or PE-based packaging to replace them with PLA-based packaging [22]. Coca-Cola and PepsiCo included the use of Bio-PET in their production line of bottles for cold drinks [28]. The Heinz company introduced its Bio-PET-based tomato sauce bottles to the market. The use of PLA in the production of flexible bags for storing frozen potatoes was promoted by the McCain company in Australia. Starch-based bioplastics were included by the Iper supermarkets and Co-op companies for the storage of organic tomatoes. The bioplastic with the greatest market penetration is PLA; companies such as Delhaize, the Asda retailer, and Danone use it in salad trays, bags, or films for tea, yogurt, and fresh food containers [74]. PLA is also used in bone splints and surgical sutures; and PHB is used in compost bags, consumer bags, and containers [18,199]. Many of these products are well established in the market; however, other polymers have been produced as demonstrations, but when presented by large companies, they were considered a promising contribution to the environment and users [200]. On the other hand, several edible bioplastics have been developed and are available in various markets, including personal care items, cosmetics, packaging, and pharmaceuticals, among others. Companies like Skipping Rocks Lab develop packaging materials based on algae; their product Ooho, is a natural packaging that biodegrades in 4–6 weeks and is even edible [201]. Lactips is a company that produces water-soluble and biodegradable thermoplastic pellets based on milk protein [202]. Candy Cutlery develops edible utensils made with 100% natural sugar [203]. Notpla is a company dedicated to the production of biodegradable and edible packaging for Notpla paper, food oil pipettes, laundry sachets, dry food sachets, bath oil sachets, food containers, rigid cutlery, and energy gel pods [204]. B’Zeos produces products such as drinking straws and packaging films. The company has raised 1.6 million euros from the development of algae-based films for packaging [205].

Although several companies are venturing into the production of bioplastics, there are certain limitations, such as high manufacturing costs, which depend on the raw material and the extraction process. As a result, the retail prices of bioplastics are often higher compared to conventional plastics. In a world where economics plays a key role for consumers, this cost increase generates rejection towards bioplastics [23]. To encourage the adoption of bioplastics as an alternative to traditional plastics, companies need to invest in new technologies. However, this principle is not yet fully applied in the bioplastic industry. Consumers do not buy products just because they are environmentally friendly; they buy them based on quality and price. Moreover, some consumers are skeptical as to the word biodegradation [140]. For example, Cereplast, a company focused on the development of biodegradable products and a TPS manufacturer, has estimated that the price of oil will reach a value of 95 dollars per barrel. This would increase the production costs of conventional plastics, promoting at the same time a growth in bioplastics demand due to the decrease in production costs. In addition, technological advances will allow a reduction in the price of bioplastics, and greater consumer education will increase their acceptability [206].

**Table 3:** Applications of bioplastics across different industries.

Type of Bioplastic	Packaging Industry	Healthcare	Cosmetics and Personal Care	Consumer Package Good	Electrical and Electronic Industry	Biophotonic Applications	Architecture and Construction	Agriculture	References
Cellulose-based	Food packaging	Coating drugs and capsules	Cosmetic cases	Headphones, eyeglass frames	Photovoltaic cells, sensors	-	Window frames and doors	Biodegradable plastic mulches	[2,82,207,208]
Lignin-based	Food packaging films	Biomedical materials	Protect skin from radiation	-	-	Photons and light to image, biophotonics	Window frames and doors	Mulching and packaging	[73,207,209]
Chitosan-based	Packing for fruit and vegetables, coatings and aerogels	Biomedical materials	Cosmetic containers	Bags, egg boxes, packaging					[198,210,211,212]
Starch-based	Packing for fruit and vegetables	Surgical films, gloves	Cosmetic containers, shampoo bottles, beauty masks	Bio-based bags, egg boxes, packaging of electronic devices				Biodegradable plastic mulches	[44,140,207,208,213]
PLA-based	Food packaging	Porous scaffolds, orthopedics, drug carriers	Cosmetic field	Bags, egg boxes, packaging of electronic devices	Photovoltaic cells, sensors		Window frames and doors	Plastic films, bags	[214,215]

In the case of bioplastics, different regulations are used to certify whether they are compostable and/or biodegradable in a given environment and under defined conditions. Companies require these certificates in order to label them as a bio-based material, but as previously discussed, being bio-based does not guarantee being biodegradable. However, the goal of a company is usually to convey to the customer the idea that the product is not of fossil origin. These aspects inhibit the growth of bioplastics in the market because the process behind obtaining the bioplastic is not considered, which can be simple or complex but directly affects the cost of the product [216,217]. In addition, companies developing new bioplastics must invest in obtaining certifications that guarantee the sustainability of the product. That is, obtaining a label generates an additional cost for the company, such as buying and using specialized equipment, and sometimes the tests take too much time. Regulatory bodies for this type of product include the International Organization for Standardization (ISO), the European Committee for Standardization (CEN), and the American Society for Testing and Materials (ASTM). However, each organization has a different concept of biodegradation, so tests often differ in processes, results, and costs [218]. For example, in Europe, the ASTM D6866 [219] standard is used, to measure the organic carbon content of biological origin in a product using an isotope quantification method. It is an analytical technique with a high level of reliability and reproducibility. However, it is not standardized for the entire world; each country chooses the certification it wants to use [216]. There are various standards to determine the biodegradation rate of the material. However, these are reported according to the environment (aquatic, soil, compostable, and industrial composting). For example, the ASTM D6691 [220] standard determines the aerobic biodegradation rate of plastic materials exposed to a population of aerobic marine microorganisms existing in natural seawater. Another example is the EN 13432 [221] standard, which is tested in various environments (aquatic, soil, home composting, and industrial composting), but with different specifications. This standard requires final biodegradability tests, compostability tests, ecotoxicity tests for plants, and a chemical characterization. This type of analysis makes it possible to identify compostable and non-compostable packaging [222]. In terms of industrial compostability standards, they are based on ISO 14855, ASTM D 5338 and/or EN 14046, and ISO 16929 and/or EN 14045 [223,224,225,226,227] standards for 90% disintegration in 12 weeks and a 90% conversion of carbon to CO_2_ at 58 °C in 6 months. The consumer must have the necessary knowledge to distinguish between a biodegradable and a compostable product. Products that achieve certification must be used specifically for the environment biodegradation that will occur, but not be thrown into a landfill [115].

In an ideal scenario, bioplastics would have their main source in renewable resources, their manufacturing process would be efficient and profitable enough to be scaled up to an industrial level, and the final product would have an adequate recycling system. These parameters would allow compliance with the principles of a circular economy [182]. The circular economy tells us that we must change the current linear system to one that allows for increased efficiency in the use of natural resources, transforming waste into resources, and implementing a new approach to production and consumption to minimize waste production and pollution [228]. Currently, bioplastics can be recycled using the different recycling techniques described in the previous paragraphs. However, the lack of a classification of waste, the inadequate disposal of the same, and the lack of environmental awareness on the part of the consumer causes this recycling system to be inefficient. Bioplastics can be recycled using the different recycling techniques described in previous paragraphs. Bioplastics play a crucial role in the bioeconomy, a renewable part of the circular economy. According to the European Commission, waste must be able to be transformed into valuable products again. This will not only help restore ecosystems by reducing plastic pollution but will also make it possible to achieve sustainable development goals, including land degradation neutrality [229]. In the case of bioplastics, the conversion of organic waste, residues, and food waste into valuable and safe bio-based products requires ensuring that this waste will not harm the producer or the land or cause overexploitation. This contributes to reducing greenhouse gas emissions by promoting more efficient primary production practices [193].

## 7. Future Perspectives and Challenges in the Development of Bioplastics

For the future, bioplastics are ideally expected to have an overall superior development and performance compared to conventional plastics, based on effective research, agile innovation, healthy and continuously growing markets, mindful and conscious use, recycling, and upcycling. However, nowadays, the production of conventional plastics is well developed, much larger, and still has substantially lower costs than that of bio-based plastics. From the technical point of view, a first challenge is in the development of bioplastics and biocomposites with excellent mechanical and thermal properties, oxygen permeability, gas barriers, and water vapor transmission rates [12]. This demands a better understanding of the relationships between the materials’ performance and their formulation, structure, processing conditions, shape, and application, as well as the stages in their lifecycle. Even though the knowledge base about their mechanical and thermal properties is ample, there are several elements that need to be studied more profoundly. These include the interactions between the different components in the formulations of bioplastics currently developed. Understanding the effect of the formulation on the temperature-dependent properties of the material in bulk would benefit from a greater use of analytical techniques such as thermogravimetric analysis and differential scanning calorimetry. Spectroscopic techniques such as Fourier-transform infrared (FTIR), UV–Visible light, and fluorescence can be used to highlight variations in the building blocks of the bio-based polymer structures.

Understanding the environmental impact of bioplastics, both in the final product and at the end of their useful life, is of vital importance. To do so, it is necessary to establish various methodologies for obtaining quantitative and qualitative indicators. The negative effects generated by bioplastics are lower than those produced by conventional plastics; however, research about the effects of their formulation on biodegradation and degradation kinetics in different conditions, and the effect of degradation products in aquatic and terrestrial environments, among others, is required. According to Naracic et al., between 1.5% and 4% of the plastic produced in the world reaches the ocean annually. The accumulation of plastic waste, microplastics, and other toxic products directly affects coral reefs, marine mammals, and terrestrial life [4]. This illustrates why one of the primary objectives of a bioplastic is its ability to biodegrade. However, as described in previous paragraphs, there is no guarantee that a bio-based plastic has this property. Biodegradation depends on the source of the raw material and the environmental conditions for degradation to occur, meeting the objective of decomposing in a relatively shorter time [21]. The use of analytical techniques will provide information by subjecting the bioplastic or biocomposite to various degradation processes [190]. There are several other topics that have received little or no attention. For example, cellulose esters are biodegradable materials obtained from renewable sources. The processing of these materials generates a considerable amount of waste that ends up in landfills [190]. Methods such as high-performance liquid chromatography (HPLC) have shown that these materials undergo gradual decomposition, resulting in the production of CO_2_ and methane. Therefore, it is increasingly urgent to develop analytical methods and techniques that allow the monitoring of these substances in real environments, and to overcome the current limitations to produce viable bioplastics for the market.

On the other hand, it must be understood that bioplastics are an alternative to conventional plastics, but they do not solve the problem of pollution. It is also necessary to educate consumers and governments to build effective infrastructure for collection, recycling, and composting. In terms of recycling, these products must have the capacity to be disposed of in accordance with the regulations established by different regulatory bodies, which must have the capacity for large-scale biodegradation, and the by-products of biodegradation can be returned to the environment without causing any negative effects [230]. PLA is one of the leading bioplastics on the market; however, it is only composted industrially (higher temperatures than those found in marine and terrestrial environments), meaning that no biodegradation occurs in domestic compost bins. This limitation means that the PLA market does not expand on a larger scale and, as it is not efficient, it must be taken off the market. Therefore, the lack of knowledge of the various composting techniques leads to poor waste management [22].

The growth of products based on chitosan, cellulose, lignin, and starch is limited by the availability of resources and the manufacturing process, which is usually not very environmentally friendly. Green technologies for the modification of these sources represent a great challenge in terms of economic viability. Price estimates for the process, source, and additives have not been established adequately, and this is evident in several studies analyzed. It should be considered that the source for obtaining cellulose, lignin, starch, and chitosan directly affects the final price of bioplastics. On the other hand, an improvement in chemical technologies and processes will reduce marginal production costs and limit price increases. However, these improvements imply the investment of capital greater than that already established. This limitation causes several companies to choose to maintain their production of conventional plastics [231].

Another challenge that bioplastics face is the high cost at which they are marketed compared to conventional plastics. This is due to their limited production, the precursors for the development of bioplastics, and their limited mechanical and thermal properties. Bioplastics cannot compete effectively with conventional low-cost plastics due to deficiencies in their formulation, processing, and end-of-life disposal. Although cheap bioplastics are available, they have not yet managed to completely displace petroleum-derived plastics, mainly due to economic reasons for both the company and the consumer. In addition, bioplastic manufacturing processes are often less energy efficient compared to traditional plastics. The additive manufacturing of biocomposites reinforced with natural and recycled fillers represents an alternative for producing short runs of parts demanding higher mechanical performance and energy efficiency [232,233,234]. Although the use of bioplastics can reduce environmental burdens, the complete transition from petroleum-derived plastics to bioplastics could generate new environmental burdens associated with agriculture and competition for food resources [22,200]. Finally, it is worth considering that complex behavioral change interventions have proved effective in promoting sustainable behaviors to tackle the challenges posed by plastic pollution. In this sense, technological innovations, economic incentives, and policy changes should be coupled with individually driven solutions to mitigate the problems associated with plastics [161,235].

## 8. Conclusions

Bioplastics are presented as a sustainable alternative to address the problem generated by plastics. In this review, the formulation of bioplastics is analyzed to understand the various interactions that occur and evaluate their potential. Analyzing these formulations helps to identify additives that can improve the mechanical and thermal properties of bioplastics, although it can also lead to complications at the end of their useful life. Conventional techniques for processing thermoplastics allow for efficient production, creating an opportunity for bioplastics to enter the plastics market at a competitive level. However, each processing technique is aimed at different applications; therefore, it is necessary to consider the context of the application, to ensure that costs and energy consumption are reduced. The biodegradation mechanism of bioplastics gives rise to different interactions between the product and the environment, so several components are involved in this process, which increase or decrease degradation. Furthermore, commercializing most bioplastics requires maintaining a consistent product quality and producing at scale at low cost, which remains a significant challenge. This problem could be solved by using agro-industrial residues as a raw material for the industrial production of bioplastics, employing an efficient processing technique, and promoting active government policies in the consumption and final disposal of these products.

## Figures and Tables

**Table 1:** Examples of the main additives used in the formulation of bioplastics, and their effect during processing and biodegradability.

Plastic/ Bioplastic	Formulation		Properties				Results and References
	Additives	Manufacturing Process	Tensile Strength/Elongation at Break	Thermal Stability	Water Permeability	Biodegradability	
Polypropylene/Polyethylene		Extrusion, compression, injection molding	High/High	High	Low	Low	[33,34]
Acetylated starch reinforced with cellulose fibers	Glycerol	Extrusion, compression molding, and injection molding	Increases/Increases	High		High	High biodegradation in soil burial due to the extraction of lower-molecular-weight compounds such as glycerol, sugars, and starch oligomers [35].
Snail shell nanoparticles and sugarcane bagasse cellulose fibers on polylactic acid (PLA) bioplastic	Acetone and PVA	Solvent casting		High	Low		The presence of acetone catalyzes against the bioplastic water absorption rate, resulting in low water permeability [36].
Thermoplastic starch and microcrystalline cellulose	Glycerol plasticizers	Extrusion and hot pressing	Increases/Decreases	High	High		High compatibility between starch matrix and MCC filler [37].
Cassava starch-based bioplastics	Glycerol	Blended and hot pressing	Increases/Decreases	Low			The interaction of glycerol and starch results in stronger and more rigid structures, improving the shelf life [38].
Ginger and green tea	White vinegar and glycerin	Solution casting	Increase/Decreases	High		High	Ginger is highly bioactive, increasing the biodegradability of any product [39].
PLA and crude palm oil	Glycerol	Solution casting	Decrease/Increases	High			Films are less soluble because of intermolecular attractions within the matrix and cross-linking [40].
Cellulose acetate (CA) prepared from Parthenium hysterophorus weed	Polyethylene glycol 600 (PEG600) ranging	Solution casting	Decrease/Increases		High	High	Plasticizers in the film cause a disarrangement of the polymer networks; as a result, there is an increase in flexibility and a reduction in tensile strength [41].
Starch and cellulose nanofibers	Stearic acid and glycerol	Extrusion and compression molding	Increases/Decreases	High	High	High	Nanofiber decreases melt flow index and hydrophilic nature [42].
Cellulose nanofiber (CNF)-reinforced PLA	Acetone and chloroform	Master batch, extrusion and injection molding	Increases/Decreases	High	High		Dispersion of fibers is adequate in PLA when the cellulose content is low [43].

## Data Availability

No new data were created or analyzed in this study.
